# Factors affecting conception rate after the first artificial insemination in a private dairy cattle farm in North Algeria

**DOI:** 10.14202/vetworld.2020.2608-2611

**Published:** 2020-12-09

**Authors:** Samir Souames, Zahra Berrama

**Affiliations:** Laboratory of Animal Health and Production, Higher National Veterinary School, Issad Abbes, Oued Smar Algiers, Algeria

**Keywords:** artificial insemination, calving to conception interval, conception rate, dairy cow, season, waiting period

## Abstract

**Aim:**

This study analyzed risk factors influencing the conception rate at the first artificial insemination (CR1) in dairy cows reared in the plain of Mitidja, which is considered a major dairy region in North Algeria.

**Materials and Methods:**

A total of 1054 lactations were used in the relational study of fertility conducted using the multivariable logistic regression model using the odds ratio (OR).

**Results:**

The breeding season had a specific effect on fertility; the first service was often followed by pregnancy when performed during autumn (AUT) (OR=1.67, p<0.05) and spring (SPR) (OR=1.65, p<0.05). The chances of obtaining conception during the first service increased significantly for a waiting period (WP) (interval between calving and time to first service) of 50-100 days postpartum (DPP) (OR=1.67, p<0.05).

**Conclusion:**

From this study, it can be concluded that no specific effect was observed relative to the breed and parity. Furthermore, CR1 significantly increased after summer calving when the first services were performed during SPR and AUT and a WP after 50 DPP.

## Introduction

In the past decades, Algeria was being confronted with a problem of dairy product shortage, with an estimated consumption for each individual of 139 L/year, the country had a supply, which provided only 40% of its needs. As a result, for many years, there was a massive importation of dairy products, which was approximately worth 500 million dollars in 2018. The volume of importation of this essential product increased by 12% in 2018 compared to the previous year [1]. Also, many breeds of dairy cattle (Holstein [H], Montbeliarde [MB], and Fleckvieh [FV]), represent only 30% of the national dairy livestock which is approximately 1 million head of cattle, despite the efforts made by the authorities. It produced an average milk yield of only 4500 kg per cow in 305 days.

The contrast between the genetic dairy potential of imported animals and their production was probably not only due to inadequate food intake but also the consequence of poor reproductive performance in these animals. Several studies mostly conducted in farms of Algeria have confirmed an increase in the interval between calving and conception to be 125 days [2], 130 days [3], and 147 days [4], and a decrease in conception at the first insemination of 25% [5] and 34% [6]. In these past decades, several authors have also reported lower conception rates at the first insemination and an increasing trend in the interval between calving and conception as well as services per conception in dairy cattle [7-9]. Some factors, more inherent to the female and its environment, can explain this decline in fertility. These include the parity [10], breed [11], waiting period (WP) [12], milk production [13], calving [14], and breeding seasons [15].

The objective of the present study was to evaluate risk factors associated with conception rate at the first artificial insemination (AI) in dairy cows reared in the plain of Mitidja, North Algeria.

## Materials and Methods

### Ethical approval

Data used in this study were collected from breed registers, individual identification cards of cows, barn schedules, and AI record sheets of a private dairy farm. Hence, there is no need for ethical approval.

### Study period and location

The research was conducted from January 2015 to October 2015, in the Mitidja area, located in the North Center of Algeria (36° 36′ N, 3° 00′ E) which is about 40 km from Algiers.

### Cows, housing, and feeding

This study was conducted at a private dairy farm housing approximately 700 milking cows aged between 3 and 12 years. The rolling average milk yield for the herd was 4500 kg per cow in 305 days.

The plain of Mitidja is a vast agricultural plain of 1400 km^2^, specialized in cultivation and dairy cattle breeding. It is characterized by a temperate Mediterranean climate with hot and dry summer (SUM) and relatively cold and wet winter (WIN), with an average monthly temperature of 23°C (16-26°C) and a pluviometry comprised between 600 and 900 mm/year.

All cows were kept under identical conditions of feeding and reproductive management. They were tied to stalls and exercise was allowed in a large paddock and was fed a total mixed ration, which comprised 40% forage (sorghum, berseem, maize silage, and alfalfa hay) and 60% concentrate (maize, barley, and wheat bran), and a commercial concentrate for lactation with trace minerals and vitamins. Data relative to the identification, breed, the number of lactation, dates of births, dates of inseminations, and calving, were retrospectively collected between 2001 and 2012 from breed registers, individual identification cards of cows, barn schedules, and artificial insemination record sheets.

### Determination of reproductive parameter

- Conception rate at the first AI (CR1): Defined as the total number of cows/heifer pregnant after the first AI divided by the total number of first insemination x 100.

### Determination of dependent and independent variables

The study of risk factors responsible for infertility shows the accurate determination of the dependent variable linked to fertility (CR1). Thus, all cows inseminated at least once and confirmed pregnant were selected for this study. The effect of five independent variables on the dependent variable generated was analyzed. Cows were divided into four groups of lactation comprising the first-calf heifers (L1) and cows in the 2^nd^ (L2), 3^rd^ (L3), and 4^th^ lactation (L4). Three groups of breeds were used in this study: H, MB, and FV.

The registered calving and the first inseminations performed have been, respectively, divided into calving season (CS) and season of first breeding (SFB). According to climatic variations of the study area, regardless of fodder availability, the females were grouped into four seasons: Autumn (AUT) from September 21 to December 20, WIN from December 21 to March 20, Spring (SPR) from March 21 to June 20, and SUM from June 21 to September 20. The intervals between calving and the first AI (WP) were divided into three classes: <50 days postpartum (DPP), 50-100 DPP, and >100 DPP.

### Statistical analysis

A relational study of fertility was conducted using a multivariable logistic regression model (method of Hosmer–Lemeshow, 1989). A binary analysis (odds ratio [OR]) was performed between a dependent dichotomous variable Y (CR1) with two values: 0 (non-achievement at AI1) and 1 (achievement at AI1) with different independent explanatory variables X: Breed, the number of lactation, CS, SFB, and WP. The comparison was assessed using the reference value (OR=1). Thus, any independent variable with a lower percentage of CR1 was selected as a reference value (Ref.). The interpretations of OR>1 or OR<1 (relative to the reference value) denote an increase or decrease in the probability of achievement at AI1, respectively. The significance of the OR was tested using the value of P at the threshold of 5%.

After the suppression of non-significant variables, the multivariate logistic regression model was applied: Logit (p) =a+b1_X1_+b2_X2_+…bn_Xn_

Where:

- a: Constant

- b: The logarithm of the OR measuring the relationship between fertility (CR1) and explanatory variables X.

The significance was tested using the value of the Wald test.

## Results

Three risk factors were identified (Table-1): CS, the SFB, and WP. The breed and parity did not have any significant effect on fertility (p>0.05). The CS and SFB increased the CR1. In comparison with other CSs, the females calved in the SUM had more chances of being fertilized at their first AI (OR=1.49, 95% CI 0.96-2.32 p=0.07). In comparison with breeding in the SUM and WIN periods, the first inseminations performed in the SPR and AUT had a good chance of initiating pregnancy (OR=1.67, 95% CI 1.11-2.51 p=0.01) and (OR=1.65, 95% CI 1.06-2.57 p=0.02), respectively.

**Table-1:** Multivariable logistic regression model for the association between risk factors and the proportion of cows that to become pregnant at first AI.

Factors	Variables	Fertility for the cow (1054)			

		CR1%	OR	CI (95%)	p-value
Breed	FL	44% (109)	Ref.		
	MB	51% (285)	1.1		NS
	H	54% (660)	1.1		NS
Parity	L1	52% (427)	1.1		NS
	L2	53% (303)	0.9		NS
	L3	49% (231)	Ref.		
	L4	54% (93)	0.8		NS
Calving season	SPR	45% (254)	Ref.		
	SUM	57% (249)	1.49	0.96-2.32	0.07
	AUT	54% (244)	1.3		NS
	WIN	51% (307)	1.2		NS
Season/AI1	SUM	44% (234)	Ref.		
	AUT	54% (246)	1.65	1.06-2.57	0.02
	WIN	56% (235)	1.3		NS
	SPR	54% (339)	1.67	1.11-2.51	0.01
WP (DPP)	<50	36% (157)	Ref.		
	50-100	50% (391)	1.64	1.03-2.60	0.03
	>100	58% (506)	1.4		NS

CI=Confidence interval at 95% NS=Non-significant on three-fold 0.05. Hosmer–Lemeshow test Chi-square=3.92 (p=0.86)

Extending the WP also had a significant effect on fertility. In comparison with the period between calving and the 49^th^ DPP (lower CR1), the chances of obtaining a conception after AI1 increased significantly between 50 and 100 DPP (OR=1.64, 95% CI 1.03-2.60 p=0.03). After 100 DPP, the WP had no specific effect on fertility.

## Discussion

This study characterized factors influencing the fertility of dairy cattle in North Algeria. Fertility, which was expressed as services per conception and conception rate at the first AI (CR1), was 1.9% and 52%, respectively. Moreover, in East Algeria, the success rate recorded in the first service conception was evaluated at 64%, with 1.3±0.9 services per conception [16]. The relational study conducted using a multivariable logistic regression model revealed that parity had no specific effect on fertility (p*>*0.05). This lack of effect has also been reported by Lane *et al*. [9].

However, in primiparous cows, Khan *et al*. [17] reported a significantly increased (p≤0.02) conception rate (parity 2, 73.7% and parity 3, 75%) than in pluriparous cows (parity 5, 40% and parity 6, 37.5%). Unlike, the number of inseminations per conception was significantly less (p<0.05) in pluriparous cows [10]. The CS had a specific effect on fertility. Compared to SPR, the conception rate at the first insemination was high during SUM calving (OR=1.49 p=0.07). Calving during the dry season had a positive effect on fertility because females can benefit from nutritional conditions and satisfy their maintenance requirements for the next wet season [18]; also, the higher first service conception rate in cows calved in SUM might have occurred since these cows were inseminated in AUT (OR=1.65 p<0.05). In this study, the season of the first insemination after calving caused significant differences in CR1, with the SUM being the least favorable period. Furthermore, Keskin *et al*. [19] concluded that high ambient temperatures (heat stress) may adversely affect fertilization and embryo survival. The hot SUM periods were the reason for a low oocyte [20] and embryonic quality [21], which were due to the high deterioration of granulosa and theca cells, thus reducing the steroidogenesis and production of progesterone by the *corpus luteum*.

According to this study, the breed has no specific effect on fertility. However, a similar study conducted in North Algeria showed a significant increase in CR1 for the H in comparison to the MB (35% vs. 28%, p<0.05) [22]. Furthermore, Toledo-Alvarado *et al*. [11] reported that the Simmental and Alpine Grey breeds had better fertility than the H and Brown Swiss dairy cows (51% and 61% vs. 48% and 49%); this difference is only partly attributable to different milk yields. Several authors have reported a negative correlation between CR1 and dairy production for the H [23,24]. Lengthening of the WP significantly increased the chances of pregnancy. Thus, in this study, CR1 increased significantly from 36% (<50 DPP) to 50% (50-100 DPP) (OR=1.64 p<0.05). After 100 days of lactation, the WP had no specific effect on fertility (p>0.05). An increase in the pregnancy rate from 47% (<100 DPP) to 69% (<150 DPP) has also been reported by Ansari-Lari *et al*. [7]. Furthermore, Polish studies by Siatka *et al.*, 2017 [25] showed that early inseminations of cows after calving (<60 DPP) resulted in a higher number of services per conception than those made between 151 and 180 DPP (2.41 vs. 2.1 p<0.0001). Several authors have reported the consequences of energy deficit on fertility postpartum [26].

An additional 1.5 points loss in body condition between calving and 30 DPP [27] or a delay in uterine involution or endometritis occurring between 20 and 33 DPP [28], also significantly affected the conception rate after calving.

## Conclusion

We conclude that conception rate at the first insemination was affected by two risk factors: Seasonal variations, the first service was often followed by pregnancy when performed during AUT and SPR, and lengthening of the WP, knowing that, the chances of obtaining conception during the first service increased significantly when artificial insemination was performed between 50 and 100 DPP.

Reproductive programs to improve CR in lactating dairy cows should focus on choosing the breeding season and the postpartum period which were two risk factors and decrease reproductive performance in dairy cows born and reared under local conditions in the plain of Mitidja.

## Authors’ Contributions

SS conceived the work. SS and ZB conducted the fieldwork, designed, and supervised the study. ZB helped with the statistical analysis. SS interpreted data, drafted, and revised the manuscript. All authors read and approved the final manuscript.
